# Microscopic Authentication of Commercial Herbal Products in the Globalized Market: Potential and Limitations

**DOI:** 10.3389/fphar.2020.00876

**Published:** 2020-06-09

**Authors:** Mihael Cristin Ichim, Annette Häser, Peter Nick

**Affiliations:** ^1^”Stejarul” Research Centre for Biological Sciences, National Institute of Research and Development for Biological Sciences, Piatra Neamt, Romania; ^2^Molecular Cell Biology, Karlsruhe Institute of Technology, Karlsruhe, Germany

**Keywords:** light microscopy, electron microscopy, authentication, plant identification, food supplement, traditional medicine, herbal product, consumer safety

## Abstract

Herbal products are marketed and used around the globe for their claimed or expected health benefits, but their increasing demand has resulted in a proportionally increase of their accidental contamination or intentional adulteration, as already confirmed with DNA-based methods. Microscopy is a traditional pharmacopoeial method used for plant identification and we systematically searched for peer-reviewed publications to document its potential and limitations to authenticate herbal medicines and food supplements commercially available on the global market. The overall authenticity of 508 microscopically authenticated herbal products, sold in 13 countries, was 59%, while the rest of 41% were found to be adulterated. This problem was extending over all continents. At the national level, there were conspicuous differences, even between neighboring countries. These microscopically authenticated commercial herbal products confirm that different magnifying instruments can be used to authenticate crude or processed herbal products traded in the global marketplace. The reviewed publications report the successful use of different magnifying instruments, single or in combinations with a second one, with or without a chemical or DNA-based technique. Microscopy is therefore a rapid and cost-efficient method, and can cope with mixtures and impurities. However, it has limited applicability for highly processed samples. Microscopic authentication of commercial herbal products will therefore contribute to raise public awareness for the extent of adulteration and the need to safeguard consumer safety against the challenges of globalization.

## Introduction

Herbal products are a heterogeneous category of goods which are produced, marketed, and used around the globe for their claimed or expected health benefits. Their commercial names depend on their final declared use (i.e., medicines or foods), and the prevailing legal frameworks and regulatory requirements (Simmler et al., 2018), such that they are sold under different names, such as herbal drugs, botanical drugs, botanicals, phytomedicines, traditional medicines, herbal medicines, traditional Chinese medicines (TCMs), traditional herbal medicinal products, natural health products, or plant food supplements (Ichim, 2019).

The use of herbal medicinal products and supplements has increased tremendously over the past decades with a substantial proportion of the world’s population relying on them as element of primary healthcare (Ekor, 2014). The global market for herbal products is rapidly expanding and expected to reach 115,000 million US$ in 2020 (Raclariu et al., 2018a) while the trade of medicinal plants will continue to advance with annual growth rates of 15–25% (Booker et al., 2012). This increasing demand for plant-based products has resulted in a proportionally increase of peer-reviewed reports of accidental contamination or intentional, economically motivated adulteration (de Boer et al., 2015; Ichim et al., 2018; Simmler et al., 2018; Grazina et al., 2020; Grosu and Ichim, 2020). A global analysis of nearly 6,000 herbal products sold in 37 countries has revealed that 27% of the products contain undeclared contaminants, substitutes, or filler species (Ichim, 2019). To address this problem, DNA barcoding as powerful strategy has recently attracted considerable attention and, along with chemical methods (de Boer et al., 2015; Pawar et al., 2017; Sgamma et al., 2017), has started to enter the regulatory systems for quality control (Pharmacopoeia Committee of P. R. China, 2015; British Pharmacopoeia Commission, 2018).

However, DNA-based diagnostics of processed food products or of mixtures from different plants can sometimes be challenging (Raclariu et al., 2018b; Grazina et al., 2020), such that this principally powerful approach has to be complemented by alternative methods (Sgamma et al., 2017). Microscopy has long been used to identify herbal products in many countries, as recorded in many pharmacopoeias, because of its advantages of small amount of sample needed, speed, reliability, simplicity, and low costs (Au et al., 2009). Moreover, histochemical techniques have been used to reveal the characteristics of tissue structure and cellular features that can be used as markers for species identification (Au et al., 2009).

Since they are often taxon-specific, in some cases even down to the species level, the morphological, anatomical, and microscopic characteristics of plant species are of great value for the purposes of scientific investigation and botanical quality control (Grazina et al., 2020). These characteristics can be used to verify plant authenticity, and to detect contaminations, adulterations, and substitutions in plant products (Michetti et al., 2019). Microscopic authentication refers to the analysis of structural, cellular and molecular features of herbal products using different forms of microscopy (Tam et al., 2006). While macroscopic and microscopic examinations are easily applied to fresh whole plants or plant parts, dried products, as those typically sold on the market, are generally difficult to identify, as many useful diagnostic characteristics are lost during dehydration (Joharchi and Amiri, 2012). In addition, often macroscopic or microscopic examinations will fail because a preparation consists of multi-component powdered samples that have been processed beyond the extent that would allow morphological characterizations (Pawar et al., 2017).

Rather than to extend the rampant increase of peer-reviewed single-case reports on adulteration of commercial herbal products, this mini-review strives to give a survey on the documented ultimate real-case scenario of all: the global market of herbal products. What are potential and limitations of microscopic food diagnostics in detecting substitution and adulteration in order to safeguard consumer safety in a globalized economy?

## Methods

### Search Strategy

We systematically searched four databases for relevant peer-reviewed studies following the PRISMA guidelines (on 17 January 2020) (Moher et al., 2009). Combinations of relevant keywords, Boolean operators and wildcards were used: [(“medicinal plant” OR herbal OR botanical OR nutraceutical OR TCM) AND (microscop* OR histolog* OR morpholog*) AND (identification OR authentication OR adulteration)] for Web of Science, PubMed, and Scopus, and [(“medicinal plant” OR herbal OR botanical OR nutraceutical) AND (microscopy OR microscopical OR histological) AND (identification OR authentication)] for ScienceDirect, due to limitations imposed by the latter search engine. The option “search alert” was activated for all the databases, to receive weekly updates after the search was performed.

### Selection Process and Criteria

*Identification*: 4,406 records were identified through database searching (WoS = 998, PubMed = 846, Scopus = 2,426, and ScienceDirect = 136), and additional 174 records through other sources.

*Screening*: 2,326 records were retrieved and their abstracts screened after the duplications had been removed. After screening, 2,062 records were excluded for not reporting data relevant to the microscopic authentication of herbal products.

*Eligibility*: 264 full-text articles were assessed and screened based on the following eligibility criteria:

1. The reported samples had to be “herbal products” *sensu lato*. A wide range of commercial names was searched for and accepted for inclusion into our analysis, all falling under two main categories: medicines or foods, with claimed or at least expected health benefits.
2. The products had to be clearly allocated to a “country” or “territory” (i.e., Hong Kong).
3. The conclusion “authentic”/”adulterated” (or similar) had to be drawn by the authors of the analyzed studies. Our involvement was restricted to operations such as counting the samples, transforming percentages in absolute numbers, without reinterpreting the experimental results in any way.
4. The samples had to be authenticated with a magnifying instrument. A wide variety of instruments were accepted, from hand lenses to electronic microscope. The morphological authentication with the naked eye was excluded from our analysis.
5. The products had to have a commercial value. Studies where the analyzed samples were “collected”, obtained “cost-free”, a “gift” or “donated” by a person, institution or company, were excluded.

The set of retrieved full-text articles was further reduced by 236 that did not meet all five eligibility criteria.

*Included*: 28 peer-reviewed articles.

## Results

Our systematic literature search identified 28 peer-reviewed publications reporting the use of magnifying instruments for the successful authentication of 508 commercial herbal products. In average, the reviewed articles reported the results for 18 herbal products, the number varying from 1 product (Liu et al., 2013; Czigle et al., 2018) to as many as 86 commercial samples (Walker and Applequist, 2012). In 13 articles, the light microscope was reported as the only instrument used for authentication, being successfully used for a total of 226 products, while the remaining studies included a second magnifying instrument, from hand lenses and stereo-microscopes till scanning electron microscopes, for the analysis of the remaining 282 products. Besides, in 20 articles at least one additional technique (DNA- or/and chemistry-based methods) was used to test authenticity. Yet, in eight studies, the exclusive use of microscopy was successfully authenticating a total of 358 commercial herbal products (**Table 1**).

**Table 1:** Microscopy-based authentication of commercial herbal products.

Country/Continent	Products/authenticated species	Reference material used & vouchers deposited (Y/N)	Products (no.)			Magnifying instrument	Other techniques used	Ref.
			Total	Authentic	Adulterated			
China	Traditional herbal tea “Ku-Ding-Cha” from markets & manufacturers (intact or fragmented dried leaves or powders)/*Ilex kudingcha* C.J.Tseng, *I. latifolia* Thunb., *Ligustrum robustum* (Roxb.) Blum, *Clerodendrum fortunatum* L., *Ehretia acuminata* R.Br.	plant samples (Y)	19	19	0	light microscope, polarized light microscope	n/a	(Tam et al., 2006)
China	Radix Polygoni Multiflori (Heshouwu)/dried root tuber of *Reynoutria multiflora* (Thunb.) Moldenke	n/a	12	12	0	light microscope	TLC, HPLC	(Zhang et al., 2005)
China	traditional “Xihuangcao” herbal tea bags from retail stores/*Isodon lophanthoides* (Buch.-Ham. ex D.Don) H.Hara, *I. lophanthoides* var. *graciliflorus* (Benth.) H.Hara, *I. serra* (Maxim.) Kudô	collected reference plant baches (Y)	8	0	8	light microscope	UPLC-ESI-QTOF-MS	(Wan et al., 2016)
China	Menispermi Rhizoma from a drug store/dried rhizome of *Menispermum dauricum* DC.	n/a	1	0	1	light microscope	UPLC-DAD-MS	(Liu et al., 2013)
Egypt	slimming herbal tea products/*Cichorium intybus* L., *Urtica dioica* L., *Origanum majorana* L., *Senna alexandrina* Mill. leaves, *Glycyrrhiza glabra* L. roots, *Apium graveolens* L. fruits, *Calendula officinalis* L. flowers, *Foeniculum vulgare* Mill., *Cichorium intybus* L.	standard herbal tea mixtures prepared from collected herbs (N)	2	2	0	light microscope	HPLC, LC-MS-MS, GC-MS	(Abdel Kawy et al., 2012)
Hong Kong	*Cordyceps sinensis* purchased fermented samples and supplements (capsules)/*C. sinensis*, *C. hawkesii*, *C. liangshanensis*, *C. militaris*	collected samples (Y)	15	4	11	light microscope, polarized light microscope	n/a	(Au et al., 2012)
Hong Kong	traditional crude drug “Wuzhimaotao” (Radix Fici Hirtae) (primarily dried roots of *Ficus hirta* Vahl)/*F. hirta* Vahl, *F. simplicissima* Lour., *F. esquiroliana* H.Lév	plants (Y)	5	3	2	light microscope	n/a	(Au et al., 2009)
India	Ayurvedic crude drug “Daruharidra” (roots of *Berberis aristata* DC.) from drug markets/*B. aristata* DC., *B. asiatica* Roxb. ex DC., *B. chitria* Buch.-Ham. ex Lindl., *B. lyceum* Royle	plants (N)	10	0	10	light microscope	HPTLC	(Srivastava and Rawat, 2013)
Iran	crude raw material of herbal drugs/27 cases of herbal drugs	n/a	78	30	48	dissecting microscope	n/a	(Joharchi and Amiri, 2012)
Thailand	white “KwaoKrua” products from local markets/*Pueraria candollei* Benth.	plant leaves (Y)	7	7	0	light microscope	ARMS-PCR, HPLC	(Intharuksa et al., 2020)
Thailand	strains of Thai medicinal fungus *Cordyceps militaris* collected from different commercial farms/*C. militaris*	n/a	7	7	0	light microscope, scanning electron microscope	DNA barcoding	(Nopparat et al., 2018)
***Asia***			***164***	***84***	***80***			
Germany	“Goji” products (dried fruits)/*Lycium barbarum* L., *L. chinense* Mill., *L. ruthenicum* Murray fruits, *L. europaeum* L., *L. chilense* Bertero, *L. ameghinoi* Speg.	whole plants (Y), fruits, DNA (Y)	19	19	0	stereo microscope, light microscope	DNA barcoding, ARMS-PCR	(Wetters et al., 2018)
Germany	three bamboo teas and five fruit teas containing bamboo leaves/bamboo (*Bambusoideae*), lemongrass (*Cymbopogon*)	plants (Y)	8	4	4	stereo microscope, light microscope	DNA barcoding	(Horn and Häser, 2016)
Germany	commercial tea mixtures containing ‘Lemon Myrtle’/*Backhousia citriodora* F.Muell., *Leptospermum petersonii* F.M.Bailey	plants (Y)	4	4	0	stereo microscope, light microscope	RAPD	(Horn et al., 2012)
Germany	Holy Basil ‘Tulsi’ mixtures (mixture teas, cut leaf fragments)/*Ocimum tenuiflorum* L., *O. basilicum* L., *O. serratum* (Schltr.) A.J.Paton, *O. gratissimum* L.	plants (Y)	4	0	4	stereo microscope, light microscope	DNA barcoding	(Jürges et al., 2009)
Germany	tea mixtures/*Dracocephalum moldavica* L.	plants (Y)	3	3	0	stereo microscope, light microscope	ARMS-PCR, RFLP	(Horn et al., 2014)
Greece	powdered Ginkgo leaf food supplement purchased in local community pharmacy/*G. biloba* L.	n/a	1	0	1	light microscope	HPLC-UV, LC-MS/MS	(Czigle et al., 2018)
Turkey	leaf products from different herbal shops/*Eucalyptus camaldulensis* Dehnh., *E. globulus* Labill., *E. grandis* W.Hill	plants (Y)	10	0	10	light microscope	TLC	(Tombul et al., 2012)
***Europe***			***49***	***30***	***19***			
USA	unprocessed products containing botanicals purchased from retail outlets/*Arnica Montana* L., *Arnica* sp., *Matricaria chamomilla* L., *Crataegus* sp., *Juniperus communis* L., *Tilia* sp., *Hypericum perforatum* L., *Schisandra* sp., *Scutellaria lateriflora* L., *Illicium verum* Hook.f.	n/a	86	65	21	hand lenses, dissecting microscope	n/a	(Walker and Applequist, 2012)
USA	“buchu” commercial raw materials and finished products/*Agathosma betulina* (P.J.Bergius) Pillans, *A. crenulata* (L.) Pillans, *A. serratifolia* (Curtis) Spreeth	plants (whole/powder) (Y)	27	10	17	light microscope, scanning electron microscope	HPTLC	(Raman et al., 2015)
USA	commercial “yohimbe” raw products/*Pausinystalia johimbe* (K.Schum.) Pierre ex Beille	plant bark (Y)	12	9	3	light microscope, scanning electron microscope	UPLC-UV-MS	(Raman et al., 2013)
USA	*Hoodia gordonii* (Masson) Sweet ex Decne. capsules/*Hoodia* sp., *Opuntia ficus-indica* (L.) Mill., *Ceropegia dichotoma* Haw., *Cynanchum* sp., *Edithcolea grandis* N.E.Br., *Huernia* sp., *Orbea* sp., *Piaranthus globosus* A.C. White & B. Sloane, *Stapelia* sp., *Tridentea choanantha* (Lavranos & Harry Hall) L.C. Leach	herbarium vouchers, collected plants (Y)	3	2	1	light microscope	PCR	(Joshi et al., 2009)
***North America***			***128***	***86***	***42***			
Argentina	single ingredient herbal products (mostly fragmented)/various species	n/a	64	44	20	light microscope	n/a	(Cuassolo et al., 2010)
Argentina	herbal drugs “canchalagua”/*Schkuhria pinnata* (Lam.) Kuntze ex Thell., *Scoparia montevidensis* (Spreng.) R.E. Fr.	herbarium vouchers, collected plants (Y)	59	40	19	stereo microscope, optic microscope	n/a	(Molinelli et al., 2014)
Argentina	fine cut (tea bags) and thick cut (fragmented herbs) mixtures/21 different species	n/a	11	7	4	stereo microscope, optic microscope	n/a	(Michetti et al., 2019)
Argentina	dietary supplements from local market/*Arthrospira* sp.	n/a	1	0	1	light microscope, polarized light microscope	FT-IR, TLC, 1D-2D NMR, PLC-MS/MS	(Redko et al., 2018)
Brazil	products from drugstores/*Smilax goyazana* A.DC., *S. rufescens* Griseb., *S. brasiliensis* Spreng., *S. campestris* Griseb., *S. cissoids* M.Martens & Galeotti, *S. fluminensis* Steud., *S. oblongifolia* Pohl ex Griseb., *S. polyantha* Griseb.	roots, leaves (Y)	15	0	15	light microscope, scanning electron microscope	TLC, DNA barcoding, SSR markers	(Martins et al., 2014)
Brazil	herbal drugs “carqueja”/*Baccharis trimera* (Less.) DC.	plants (Y)	15	8	7	magnifying glass, microscope	GC-FID	(De Ferrante et al., 2007)
Peru	*Hoodia gordonii* (Masson) Sweet ex Decne. tablets (white)/*Hoodia* sp., *Opuntia ficus-indica* (L.) Mill., *Ceropegia dichotoma* Haw., *Cynanchum* sp., *Edithcolea grandis* N.E.Br., *Huernia* sp., *Orbea* sp., *Piaranthus globosus* A.C. White & B. Sloane, *Stapelia* sp., *Tridentea choanantha* (Lavranos & Harry Hall) L.C. Leach	herbarium vouchers, collected plants (Y)	2	1	1	light microscope	PCR	(Joshi et al., 2009)
***South America***			***167***	***100***	***67***			
**Total**			**508**	**300**	**208**			

**Figure 1:**
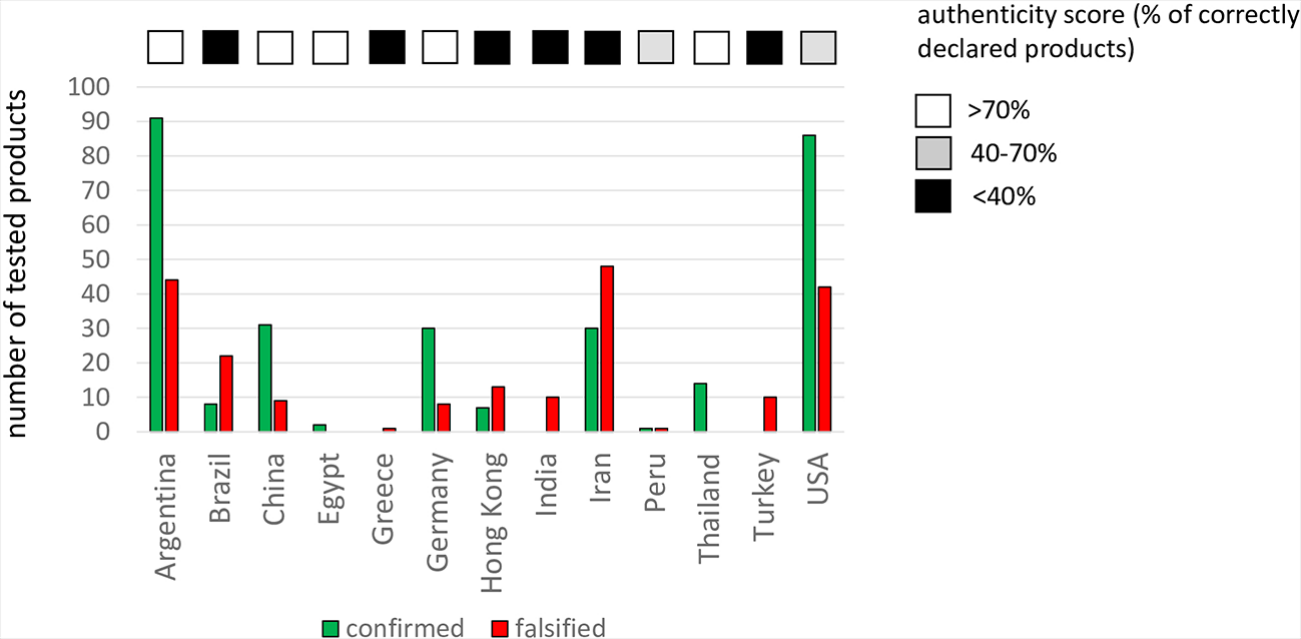
Numbers of commercial herbal products verified by microscopical diagnostics over different countries. Products, where authentication confirmed the declared content are given in green, products, where the declaration was falsified, in red. The fraction of correctly declared products in a given country is indicated by the authenticity score.

We have reviewed the authenticity results for 508 herbal medicines and food supplements traded in thirteen countries or territories (i.e., Argentina, Brazil, China, Egypt, Hong Kong, Germany, Greece, India, Iran, Peru, Thailand, Turkey, and USA), geographically representing all inhabited continents, except Australia. For two countries (i.e., Argentina and USA), more than 100 commercial herbal products have been analyzed and microscopically authenticated from their respective national marketplace. For additional six countries (i.e., Brazil, China, Germany, Hong Kong, Iran, and Thailand), more than 10 herbal products were analyzed, while for Egypt, Greece, India, Peru, and Turkey, 10 products or less were reported (**Figure 1**).

The overall authenticity of the microscopically authenticated commercial herbal products from the global marketplace was 59% (n = 300 products), while the rest of 41% (n = 208 products) were found to be adulterated (**Table 1**). All or at least most (>70%) products were reported to be authentic in Argentina (n = 135), China (n = 40), Germany (n = 38), Thailand (n = 4), and Egypt (n = 2). In the USA (n = 128) and Peru (n = 2), a substantial part (> 30%) was wrongly declared, and a third group of countries, comprising Iran (n = 70), Brazil (n = 30), India (n = 10), Turkey (n = 10), and Greece (n = 1) showed authenticity score is lower than 40%. It should be kept in mind that the numbers of samples was geographically heterogeneous: At continental level, the highest number of commercial herbal products was reported for South America (n = 167), followed very closely by Asia (n = 164), by North America (n = 128) and, distantly, by Europe (n = 49). Instead, almost half (49%) of the total products (n = 167) microscopically authenticated in Asia were reported to be adulterated, followed by South America (40%), Europe (39%), and more distantly by North America (33%).

## Discussion

Our survey on the extent of herbal adulteration as detected by microscopical diagnostics supports a previous study using DNA-based authentication (Ichim, 2019). On the global scale, almost half of herbal products did not contain, what they declared. However, there are substantial differences between the different national markets, sometimes even between neighboring countries, such as Argentina (with a high authenticity score among the 135 tested products) and Brazil (with a low authenticity score among the 30 tested products). In the following, we will discuss to what extent this startling outcome is robust against unavoidable sampling bias, and what the potential and the limitations of microscopic diagnostics are. Using two case studies, we will address possible reasons for this high degree of adulteration, and conclude by deriving some suggestions, how consumer protection can be safeguarded in times of globalization.

### Sampling Heterogeneity and Its Reasons

The frequency of adulteration in the current study exceeds that seen for the study based on DNA authentication (Ichim, 2019). This might stem from the fact that the sample size is different (DNA authentication: n = 5,957; microscopic authentication: n = 508) by a factor of ten. There are different reasons for this obvious sampling bias: to publish results from a traditional method in peer-reviewed journals is much more difficult as compared to publishing DNA-based, “new” diagnostic approaches. The countries with a functional consumer safety system might be underrepresented as the authentication results of the commercial samples screened by the respective institutions will be published in internal bulletins or protocols, rather than in peer-reviewed journals. There is a second, methodological reason, though: microscopic diagnostics requires not only patience, exact observation, and long experience, but also deep knowledge of plant anatomy and plant biodiversity. This expertise is currently lost rapidly in most countries, and may be one reason, why some countries are not represented in our sampling. The comparison of Brazil (n = 30, most adulterated) and Argentina (n = 178, most authentic) indicates a further reason for sampling bias: the lack of methodological expertise may also correlate with a lack of consciousness that there is a serious problem.

### Prospects and Limitations of Microscopic Diagnostics

Our current study clearly confirms previous results (Gao et al., 2017) that a substantial proportion of commercial herbal products are adulterated. Despite the *caveat* on sampling bias, it can also be concluded that microscopic diagnostics can detect adulterations more thoroughly than DNA-based authentication alone. The microscopically authenticated products were purchased from a wide variety of markets and shops, and were processed for quite diverse purposes. Thus, microscopic authentication is robust enough to be used along local, national, and international value chains for herbal products (Booker et al., 2012; Heinrich et al., 2019). Qualitative cellular and anatomical details, such as calcium oxalate crystals, idioblasts, xylem cells, stone cells, or stomatal complexes are well covered by monographies forming the base for pharmacopeias (Tam et al., 2006). In difficult cases, morphological identification can be supported by histochemical analysis to detect diagnostic features of the specimen (Au et al., 2009). In addition, microscopy allows to trace contamination by non-plant materials, such as insects or even inanimate matter, which would go unnoticed in a DNA-based test, such as pieces of wire, nylon, soil, stones, or sand (Cuassolo et al., 2010; Michetti et al., 2019). Despite these undisputable advantages, microscopic diagnostics also has to face several challenges: for the often exotic species, monographies on their diagnostic characteristics are not available and have to be newly established. A precondition for this endeavor is the availability of authentic reference material, either from wild or cultivated resources, Botanical Gardens, germplasm centers, or herbarium vouchers. The validation and verification of such reference materials is an absolute must. Unfortunately, a substantial fraction of these materials are mislabeled as well (Goodwin et al., 2015), because *“Rapidly increasing numbers of specimens in an increasing numbers of herbaria are not being revived because there are too few taxonomists”*. Some microscopical techniques, such as SEM, require expensive equipment, which may not be affordable in a lab. TEM in particular, requires extensive sample processing, such as fixation, embedding, ultrasectioning, and contrasting, which easily can introduce artifacts that are misleading, if the user is not very experienced. A further challenge for microscopic diagnostics are heavily processed products, such as finely cut material or ground powders. Although one can try to process the collected reference plant material in a similar manner to match the commercial products, often the diagnostic features are lost.

### Herbal Adulteration as Product of Economic Incentive and Ambiguous Nomenclature

There seem to be mainly two drivers for adulteration: (i) limited supply and high dynamics of demand; (ii) ambiguities of traditional versus scientific nomenclature. In the following, both factors are exemplarily discussed. Optical microscopy can help here to screen out in a robust and cost-effective way, samples that appear suspicious and might deserve closer scrutiny also involving other, more laborious, and more expensive, methods. The connection between limited supply, sky-rocketing demand, and the use of light microscopy can be illustrated using the *Cordyceps* case.

#### Optical Microscopy—A Cheap Technique to Operate Can Detect Adulteration in a Multi Billion Industry

The insect-parasitic fungus *Cordyceps sinensis* is widely used and valued in Asian traditional medicine (Elkhateeb et al., 2019), but endemic to a very restricted area in the Tibetan plateau (Li et al., 2011). In the meantime, it has been recognized to be not related to other members of the genus and has been renamed as *Ophiocordyceps sinensis*. During the last decade, *Cordyceps* had been discovered as “superfood” and is now amply used as supplement for food supplements. The combination of restricted habitat and increasing demand have led to over-harvesting and a dramatic rise in its price (Liu et al., 2011). The market volume of traded products declared as *Cordyceps* exceeds the annual harvest of *O. sinensis* by a factor of 20 and has reached 470 million USD in 2018. Around 70% seems to be in fact the only distantly related parasitic species *C. militaris* (GlobeNewswire, 2019). The economic gain of this adulteration is tremendous: the crude drug was already at 13,000 USD/kg in 2008, but has skyrocketed to USD $20,000 to 40,000 per kg within 5 years (Lo et al., 2013). In this context, light microscopy was successful to authenticate these products, no matter, whether marketed, as raw fruiting body (Nopparat et al., 2018), or in fermented or processed form as capsules (Au et al., 2012).

#### Traditional Nomenclature Can Cause Non-Intentional Adulteration: The Case of Bamboo Tea

Traditional nomenclature is not based on phylogenetic relationship, but on common use (Berlin et al., 1966). Globalization shifts plants out from their traditional functional context, and this represents a major source for non-intentionally adulteration, as exemplarily addressed for the product Bamboo tea (Horn and Häser, 2016), which appeared on the European market from the mid of the last decade. Bamboo belongs to the Poaceae with characteristic dumbbell-shaped guard cells. When leaf fragments of this product were investigated by bright-field microscopy, instead, kidney-shaped guard cells with two subsidiary cells, arranged in parallel rows, were seen. This diacytic stoma type was definitely not consistent with the declared Poacean ingredients, but rather pointed to a specimen of the genus *Dianthus* (carnation). This could be confirmed using the plastidic markers matK and rbcL. Different accessions from this genus were then purchased from different Botanical Gardens and commercial sources and taxonomically verified to serve as references. When the histology of completely developed leaves from these plants was investigated by microscopy, the samples could be assigned to the species *Dianthus chinensis* L. The background of this curious case of adulteration (bamboo and carnation are evolutionarily far apart) is to be sought in the problematic use of vernacular names in commercial products. The young leaves of the bamboo species *Sasa palmate* (Burb.) E.G.Camus, *Sasa kuriliensis* (Rupr.) Makino & Shibata, and *Lopaterum gracile* have a long history in TCM and are supposed to support the breakdown of body sediments including fat. They are traded under the name *Dan Zhu Ye*. Also Chinese carnation (*Dianthus chinensis* L.) is used in TCM, but for a different purpose (for instance, it acts diuretically). Since the leaves of this plant resemble young bamboo leaves, and since it is a traditional component of Chinese Stone Gardens, it is often called *Shi Zhu* (“Stone Bamboo”). In the context of TCM, it is fairly clear, what plant is handled. However, the rapid increase in consumer demand had caused bottlenecks in the producing countries. When supply is under constraint, the internet search for Bamboo tea can easily lead to the purchase of products that are branded as “Bamboo Carnation Tea”. This had happened in the current case, and again underlines, how important it is to install a systematic authentication of commercial products, preferably already in the harbors, where the material enters the industrial supply chain.

### Concluding Remarks

The use of microscopy and molecular markers as diagnostic tools for the authentication of plant food ingredients is rarely used in combination. However, this would be important, because each of the two approaches has individual advantages and disadvantages. Microscopy is a rapid and cost-efficient method, and can cope with mixtures and impurities. However, it has limited applicability for highly processed samples. It is important to keep this art alive as essential part of university curricula. It is also important to raise public awareness for the extent of adulteration and the need to safeguard consumer safety against the challenges of globalization.

## Author Contributions

MI performed the literature systematic search. MI, AH, and PN wrote and approved the submitted manuscript.

## Funding

This work was supported by a grant of the Ministry of Research and Innovation through Program 1 - Development of the National R&D System, Subprogram 1.2 - Institutional Performance - Projects for Excellence Financing in RDI, contract no. 22PFE/2018 (for MI). This publication was supported by the National Core Program funded by the Romanian Ministry of Research and Innovation, project number 25N/11.02.2019, BIODIVERS19270401 (for MI).

## Conflict of Interest

The authors declare that the research was conducted in the absence of any commercial or financial relationships that could be construed as a potential conflict of interest.

## Supplementary Material

The Supplementary Material for this article can be found online at: https://www.frontiersin.org/articles/10.3389/fphar.2020.00876/full#supplementary-material
